# Clinical effect analysis of laminectomy alone and laminectomy with instrumentation in the treatment of TOLF

**DOI:** 10.1186/s12891-021-04564-3

**Published:** 2021-08-09

**Authors:** Zhi-Wei Wang, Zheng Wang, Yan-Hong Zhou, Jia-Yuan Sun, Wen-Yuan Ding, Da-Long Yang

**Affiliations:** 1 Department of Spine Surgery, The Third Hospital of Hebei Medical University, 139Ziqiang Road, 050051 Shijiazhuang, PR China; 2 Hebei Provincial Key Laboratory of Orthopaedic Biomechanics, 139Ziqiang Road, 050051 Shijiazhuang, PR China

**Keywords:** Laminectomy, Instrumentation, Clinical effect, TOLF, Spine

## Abstract

**Background:**

To explore the clinical effect of laminectomy alone and laminectomy with instrumentation in the treatment of TOLF.

**Methods:**

A retrospective study was conducted on the clinical data of 142 patients with TOLF and laminectomy who underwent spine surgery at XXX Medical University from January 2003 to January 2018. According to whether the laminectomy was combined with instrumentation, the patients were divided into two groups: group A (laminectomy alone (LA), n = 77) and group B (laminectomy with instrumentation (LI), n = 65). Comparisons of possible influencing factors of demographic variables and operation-related variables were carried out between the two groups. In this study, the clinical effects of LA and LI in the treatment of TOLF were discussed. Thus, we explored the clinical effect of LA and LI in the treatment of TOLF.

**Results:**

In terms of demographics, there was a statistically significant difference in BMI between group A and group B (P < 0.05). The differences in age, sex, smoking, drinking, heart disease, hypertension and diabetes were not statistically significant (P > 0.05). In terms of preoperative symptoms, there was a significant difference in gait disturbance, pain in the LE, and urination disorder between group A and group B (P < 0.05), but there was no significant difference in other variables between the two groups (P > 0.05). In terms of operation-related variables, there was a significant difference in the preoperative duration of symptoms, intramedullary signal change on MRI, dural ossification, residual rate of cross-sectional spinal canal area on CT, shape on the sagittal MRI, operation time, pre-mJOA, post-mJOA at 1 year, and leakage of cerebrospinal fluid between group A and group B (P < 0.05), but there was no significant difference in other variables between the two groups (P > 0.05). The preoperative average JOA score of group A was 6.37 and that of group B was 5.19. In group A, the average JOA score at 6 months, 1 year and 2 years after surgery was 7.87, 8.23 and 8.26, respectively, and the average JOA score improvement rate was 32.79 %, 38.32 and 38.53 %, respectively. In group B, the average JOA score at 6 months, 1 year and 2 years after surgery was 7.74, 8.15 and 8.29, respectively, and the average JOA score improvement rate was 39.15 %, 46.86 and 47.12 %, respectively.

**Conclusions:**

Currently, there is no consensus on whether instrumentation is needed after laminectomy for TOLF. We found that for patients with a long duration of gait disturbance, urination disorder, preoperative duration of symptoms, intramedullary signal change on MRI, dural ossification, residual rate of cross-sectional spinal canal area on CT less than 60 %, and shape on the sagittal MRI being beak and low, pre-mJOA had better clinical effects after LI as compared to those after LA, and the incidence of perioperative complications was lower.

## Background

**Fig. 1:**
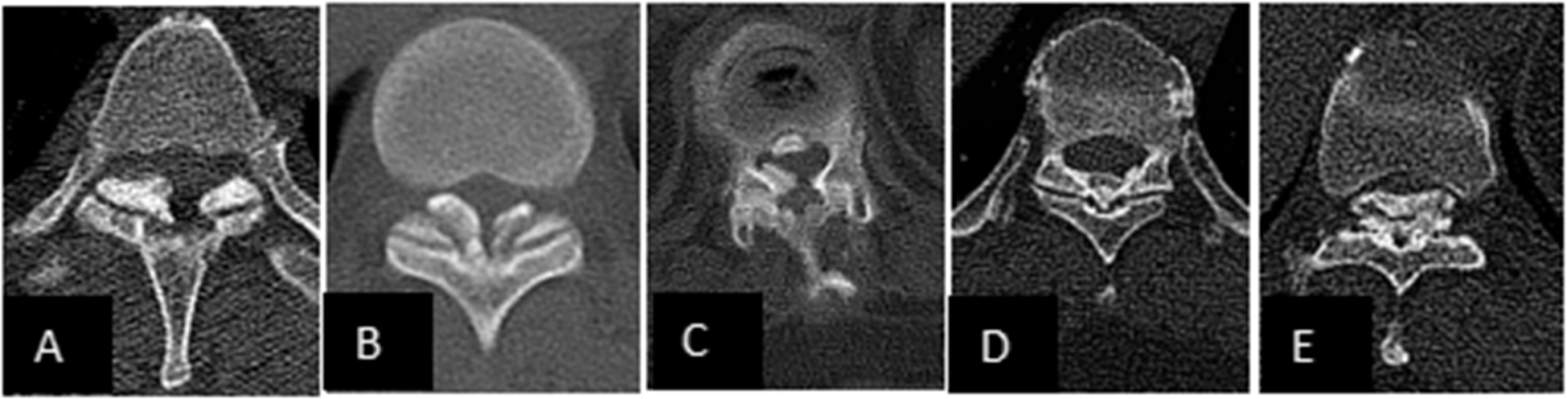
Sato’s classification of TOLF. E Sato’s classification, **A** lateral type, **B** extended type, **C** enlarged type, **D** fused type, **E** tuberous type

**Fig. 2:**
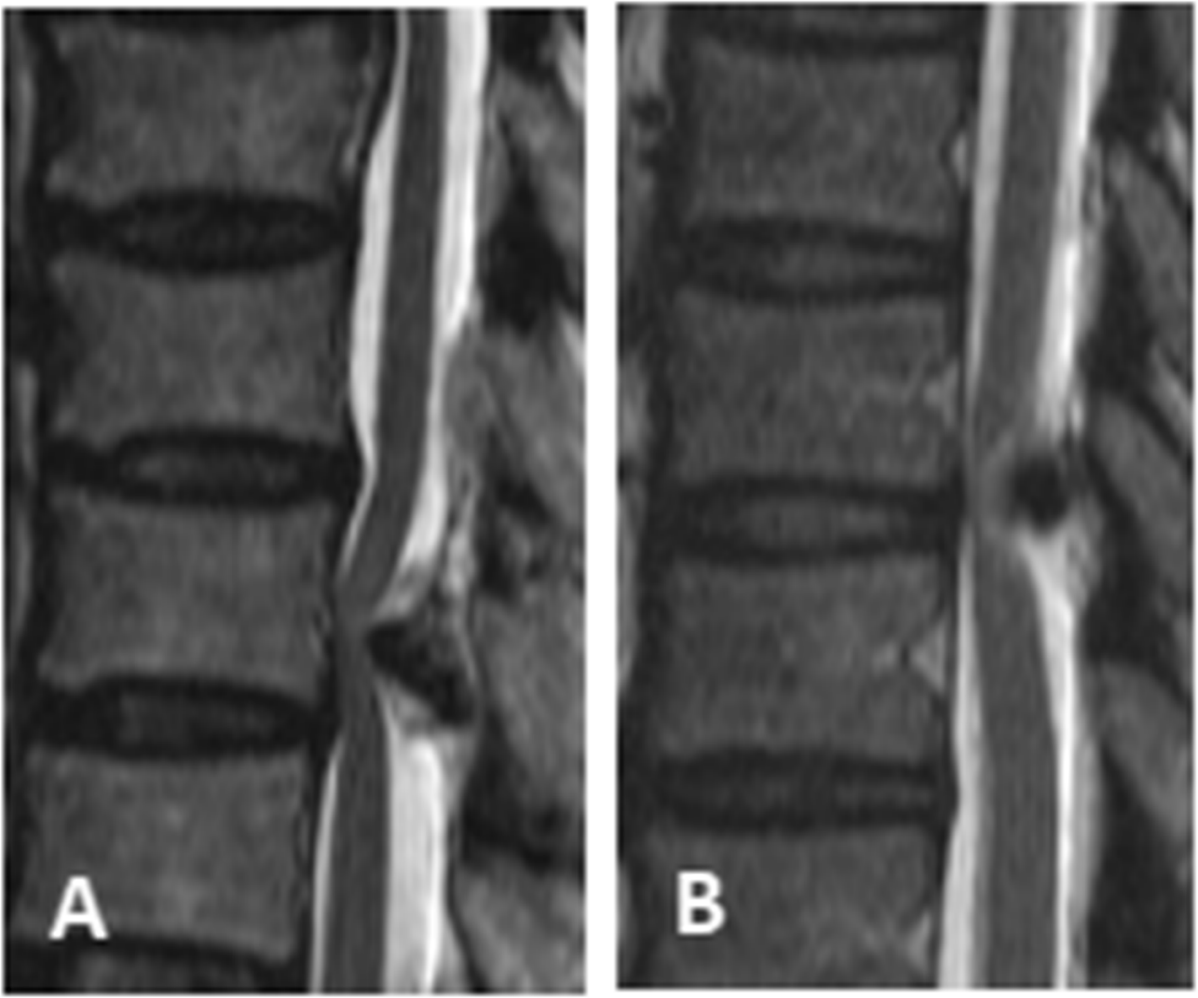
Kuh’s classification of TOLF. **A**-**B** Sato’s classification, **A** beak type and **B** round type

Thoracic ossification of the ligamentum flavum (TOLF) is a pathological heterotopic ossification. The affected thoracic vertebra is mainly concentrated in the lower thoracic vertebral area, successively T10-11, T9-10, and T11-12. Ossification often causes thoracic spinal stenosis and produces the corresponding symptoms of spinal cord compression [1–4]. It is a common cause of thoracic myelopathy [5], with an incidence of 3.8-26 % [6]. According to Sato’s classification [7], TOLF can be divided into five types: lateral type, extended type, enlarged type, fused type, and tuberous type (Fig. 1). According to Kuh’s classification [8], TOLF can be divided into two types: beak type and round type (Fig. 2). Conservative treatment of TOLF is usually ineffective. Because most of the disease progresses slowly, the thoracic spinal cord nerves have been compressed for a long time and have become flattened or even atrophied when obvious symptoms often appear. Therefore, surgical decompression should be performed as soon as possible for patients who are suspected of having TOLF [9]. Early detection, early diagnosis and early treatment are very important to the satisfaction of postoperative efficacy. Traditional open surgery includes posterior laminectomy, laminectomy and laminoplasty, among which laminectomy is widely used and has become one of the classic surgical procedures [10, 11]. However, there is no clear standard for the combined application of internal fixation after laminectomy. The purpose of this study was to explore the clinical efficacy analysis of LA and LI in the treatment of TOLF.

## Materials and methods

### Inclusion of patients

A retrospective study was conducted on the clinical data of 142 patients with TOLF and laminectomy who underwent spine surgery at the XXX Medical University from January 2003 to January 2018. Inclusion criteria included: (1) patients with TOLF undergoing laminectomy; (2) complete clinical data available; and (3) the last follow-up was more than 2 years ago. Exclusion criteria included: (1) combined with anterior disc herniation or obvious compression of the spinal cord or nerve root; (2) combined with other spinal cord-related diseases; (3) trauma, inflammation, infection, or tumour involving the spine; or (4) patients with severe compression of cervical or lumbar nerves. A total of 142 patients were included in the study (89 males, 53 females). According to whether or not laminectomy was combined with instrumentation, the patients were divided into two groups: group A (laminectomy alone (LA), n = 77) and group B (laminectomy with instrumentation (LI), n = 65). The Ethics Committee of the XXX Medical University approved the study, and written informed consent was obtained from all patients before they were recorded.

### Study variables

Variables, including age, sex, BMI, smoking, drinking, heart disease, hypertension, and diabetes, were collected by different staff at the time of admission. Preoperative symptoms (gait disturbance, thoracic and back pain, numbness in LE, pain in LE, sensory deficit in LE, urinary disorder, and sense of constriction in thoracic or abdomen) were recorded in the physical examination. Operation-related variables included preoperative duration of symptoms, OLF segment, intramedullary signal change on MRI, dural ossification, residual rate of cross-sectional spinal canal area on CT, shape on sagittal MRI, operation time, blood loss, pre-mJOA, post-mJOA, and JOA improvement rate.

### Operation method

The same group of surgeons performed the operation. The patient was in the prone position under general anaesthesia. During the operation, electromyography and somatosensory evoked potentials were monitored. The operation segment was confirmed by C-arm fluoroscopy. A posterior median incision was made along the spinous process; the skin and subcutaneous tissue were separated layer by layer; and the structures of the spinous process, lamina and articular process were exposed. For patients who underwent laminectomy with instrumentation, pedicle screws were screwed in first. Then, the spinous process and the outer layer of the lamina were bitten off, and the lateral lamina were removed by a high-speed grinding drill in the longitudinal direction at the inner edge of the facet joints of the bilateral lamina. Then, the interspace of the lamina was enlarged, and the ligaments between the upper and lower spine were removed. Then, a high-speed grinding drill was used to scan the middle lamina with normal saline flushing, and each line was not polished too deeply until the ossified tissues were eggshell-like translucent. The remaining thin layer of bone was lifted with towel forceps, and the inner vertebral plate, OLF and dural mater were gently probed with a nerve exfoliator to see if there was adhesion. If there was no adhesion, the inner vertebral plate and OLF were removed to achieve full decompression. The surgeon continued to nibble on both sides to the outer 1/3 of the facet joint until both sides of the dural sac were exposed; at this time, the dural sac could be completely expanded. In this study, 16 patients (4 cases in group A and 12 cases in group B) were found to have heavy adhesion to the dura mater, which could not be separated. They were resected together and repaired with local fascia. After decompression was completed, the patients who underwent laminectomy and internal fixation had the prebent connecting rod installed on the pedicle screw, tightened and locked. Afterwards, the operation field was thoroughly rinsed, and indwelling negative pressure drainage tube was placed, and the incision was sutured layer by layer to finish the operation. All patients were routinely treated with antibiotics within 3 days after the operation. Seven days after the operation, patients were encouraged to wear braces while walking, and brace protection was maintained for approximately 3 months.

### Satisfaction evaluation

All patients were followed up by outpatient visits or telephone visits at 6 months, 1 year, and 2 years postoperatively. Neurological function was evaluated by the Japanese Orthopaedic Association (JOA) score during the follow-up. The thoracic neurological function JOA score (out of 11) was improved compared with the cervical JOA score (out of 17), 4 points on dexterity of hands and 2 points on sensation of upper limbs were removed based on the cervical JOA score (Table 1) [12, 13]. JOA recovery rates were calculated as follows: [(postoperative JOA score - preoperative JOA score)/(11 - preoperative JOA score)]×100 %. Recovery outcome was ranked as excellent (≥ 75 %), good (50-74 %), general (25-49 %), or poor (< 25 %) [11]. During the follow-up, anterior and lateral X-rays and three-dimensional CTs were taken.

**Table 1:** Modified Japanese Orthopaedic Association (JOA) scoring system for the assessment of thoracic myelopathy. (Total score 11 points)

**Functional score**	
**Motor function: lower limb**	
0	Unable to walk.
1	Support was needed to walk on flat ground.
2	Need a cane or aid on flat ground.
3	Walking on flat ground or up stairs did not require support, but the lower limbs were not flexible.
4	Normal.
**Sensory function: lower limb**	
0	Obvious sensory impairment.
1	Mild sensory impairment or numbness.
2	Normal.
**Sensory function: Trunk**	
0	Obvious sensory impairment.
1	Mild sensory impairment or numbness.
2	Normal.
**Bladder function**	
0	Uroschesis.
1	Highly dysuria, laborious, irretention or incontinence.
2	Mild dysuria, frequent urination, hesitation in urination.
3	Normal.

### Statistical analysis

All statistical analyses were carried out by SPSS software version 22.0 (IBM, Armonk, NY, USA), and the test level was α = 0.05. The measurement data between the two groups were compared by independent sample t-tests or nonparametric tests according to whether they were in line with normal distribution and homogeneity of variance. Analysis of counting data was carried out by chi-square test. Significance was accepted at p < 0.05.

## Results

All 142 patients (89 males and 53 females) underwent successful surgery. According to whether or not the laminectomy was combined with instrumentation, the patients were divided into two groups: group A (laminectomy alone (LA), *n* = 77) and group B (laminectomy with instrumentation (LI), n = 65). All patients were followed up for 2 years or more. In terms of demographics, there was a statistically significant difference in BMI between group A and group B (*P* < 0.05). The differences in age, sex, smoking, drinking, heart disease, hypertension and diabetes were not statistically significant (*P* > 0.05) (Table 2). In terms of preoperative symptoms, there was a significant difference in gait disturbance, pain in LE, and urination disorder between group A and group B (*P* < 0.05), but there was no significant difference in other variables between the two groups (*P* > 0.05) (Table 3). In terms of operation-related variables, there were significant differences in preoperative duration of symptoms, intramedullary signal change on MRI, dural ossification, residual rate of cross-sectional spinal canal area on CT, shape on the sagittal MRI, operation time, pre-mJOA, post-mJOA at 1 year, and leakage of cerebrospinal fluid between group A and group B (*P* < 0.05), but there was no significant difference in other variables between the two groups (*P* > 0.05) (Table 4).

**Table 2:** The main demographic variables of the patients with TOLF in 2 groups

Characteristics	Group A (*n* = 77,54.2 %)	Group B (*n* = 65,45.8 %)	*P* value
Age (years)	59.85 ± 8.74	60.44 ± 6.98	0.586
Sex (male/female)	48/29	41/24	0.928
BMI (kg/m²)(≤27/>27)	52/25	32/33	0.027*
Smoking (yes/no)	44/33	35/30	0.694
Drinking (yes/no)	46/31	40/25	0.827
Heart disease (yes/no)	27/50	20/45	0.588
Hypertension (yes/no)	35/42	24/41	0.304
Diabetes (yes/no)	19/58	15/50	0.824

*BMI* Body Mass Index

*The difference possessing statistical significance

*P < 0.05

**Table 3:** The main Preoperative symptoms of the patients with TOLF in 2 groups

Characteristics	Group A (*n* = 77,54.2 %)	Group B (*n* = 65,45.8 %)	*P* value
Gait disturbance	31	42	0.004*
Thoracic and back pain	12	11	0.710
Numbness in LE	23	29	0.069
Pain in LE	29	14	0.037*
Sensory deficit in LE	56	45	0.637
Urination disorder	20	32	0.004*
Sense of constriction in thoracic or abdomen	21	23	0.298

*LE* lower extremity

*The difference possessing statistical significance

*P < 0.05

**Table 4:** The related risk factors of the patients with TOLF in 2 groups

Characteristics	Group A(*n* = 77,54.2 %)	Group B(*n* = 65,45.8 %)	*P* value
Preoperative duration of symptoms(months)	19.17 ± 10.36	26.81 ± 12.14	0.013*
OLF segment			0.987
Upper thoracic(T1−4)	7	6	
Middle thoracic (T5−8)	4	3	
Lower thoracic(T9−12)	66	56	
Intramedullary signal change on MRI			<0.001*
Yes	14	52	
No	63	13	
Dural ossification			0.007*
Yes	4	13	
No	73	52	
Residual rate of cross-sectional spinal canal area on CT			<0.001**
≤60 %	19	50	
>60 %	58	15	
Shape on the sgittal MRI			0.002*
Beak	45	21	
Round	32	44	
Operation time (min)			<0.001**
≤250	51	17	
>250	26	48	
Blood loss (ml)	493.33 ± 164.39	614.81 ± 193.56	0.013*
Pre-mJOA	6.37 ± 1.59	5.19 ± 1.78	0.010*
Post-mJOA			
At 6 months	7.87 ± 1.17	7.74 ± 1.26	0.697
At 1 year	8.23 ± 1.33	8.15 ± 1.06	0.792
At last follow-up	8.26 ± 1.05	8.29 ± 1.17	0.920
JOA improvement rate			
At 6 months	32.79 ± 20.65	39.15 ± 21.14	0.073
At 1 year	38.32 ± 21.49	46.86 ± 20.69	0.018*
At last follow-up	38.53 ± 20.72	47.12 ± 20.71	0.054
Complications			
Wound infected			0.765
Yes	7	5	
No	70	60	
Delayed wound healing			0.426
Yes	9	5	
No	68	60	
Leakage of cerebrospinal fluid			0.013*
Yes	4	12	
No	73	53	
Neurological deficit	Nil	Nil	
Recurrence	Nil	Nil	
Intercostal pain			0.439
Yes	6	3	
No	71	62	
Thrombosis of lower extremities			0.934
Yes	5	4	
No	72	61	
Others			0.426
Yes	9	5	
No	68	60	

*mJOA* Modified Japanese Orthopaedic Association

*The difference possessing statistical significance

*P < 0.05,**P = 0.000

**Fig. 3:**
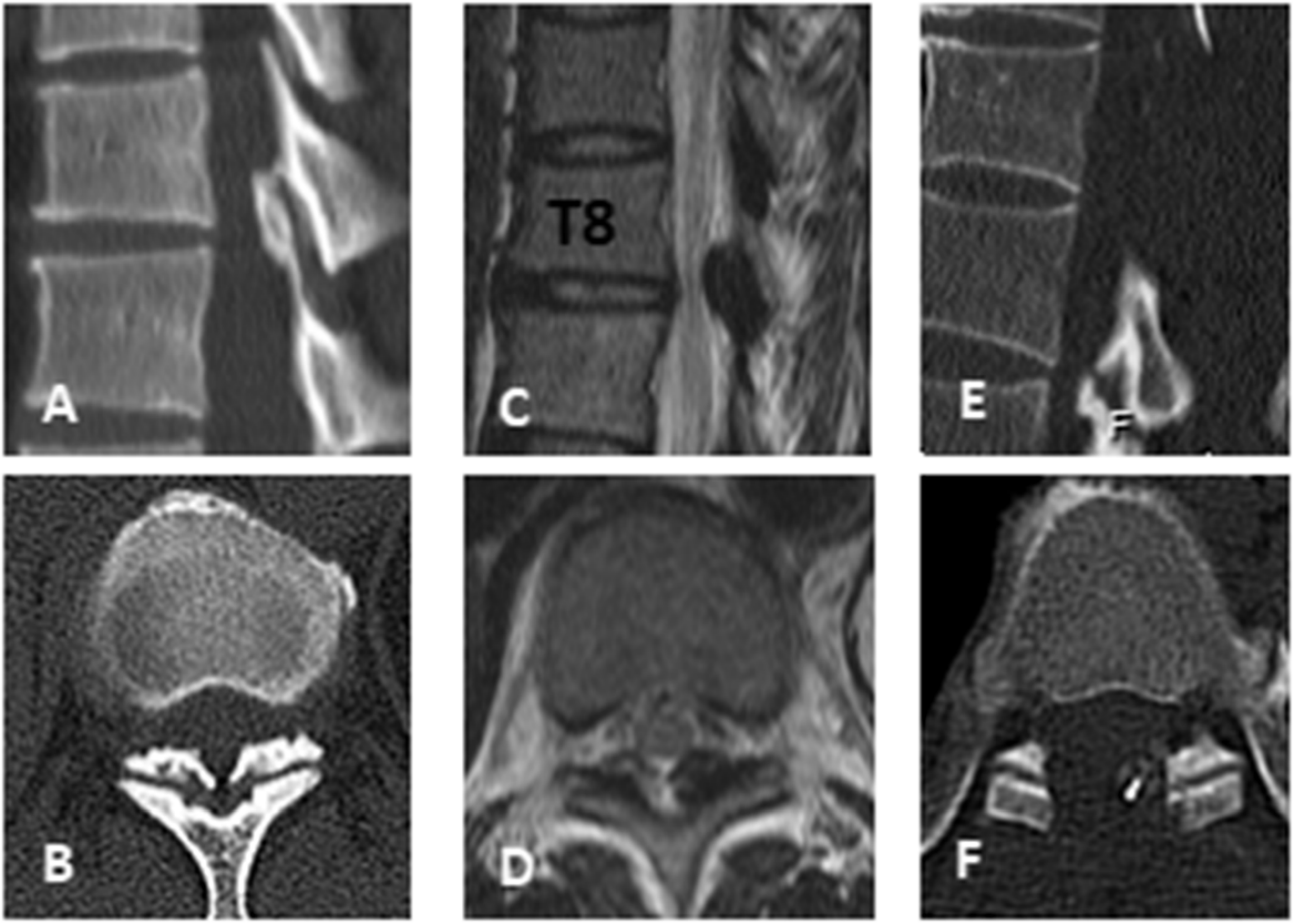
Laminectomy alone. A 55-year-old female patient, ossification of ligamentum flavum and compression degeneration of spinal cord could be seen on CT (figure **A**, **B**) and MRI (figure **C**, **D**) before operation. We performed OLF resection with LA (Fig. **E**, **F**). During the 2-year follow-up, the preoperative JOA score increased from 7 to 8.5 (JOA score improvement rate was 37.5 %)

**Fig. 4:**
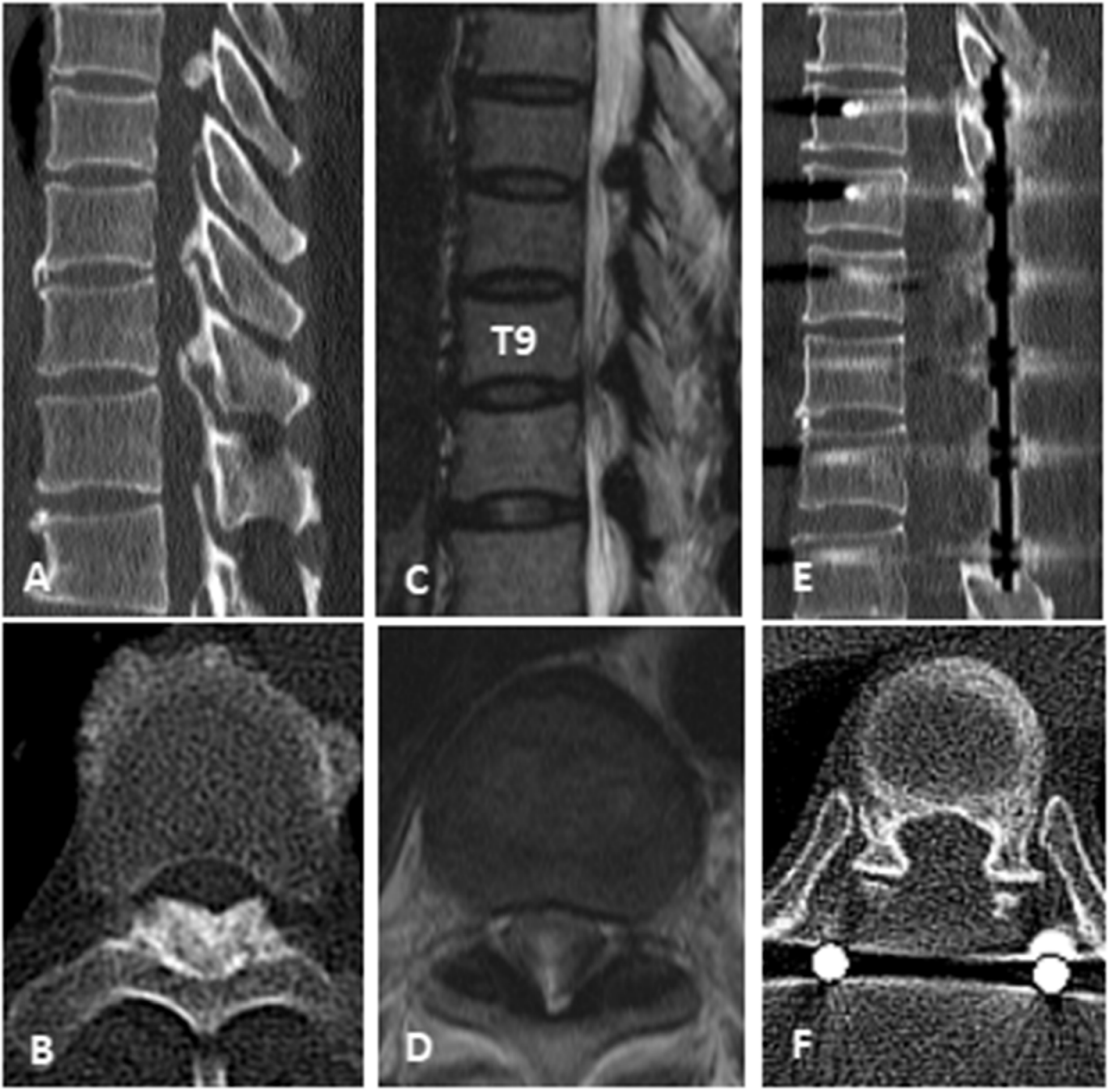
Laminectomy with instrumentation. A 62-year-old male patient, ossification of ligamentum flavum and compression degeneration of spinal cord were seen in CT (figure **A**, **B**) and MRI (figure **C**, **D**) before operation. We performed OLF resection with LI (Fig. **E**, **F**). During the 2-year follow-up, the preoperative JOA score increased from 4 to 8 (JOA score improvement rate was 57.1 %)

No neurological deterioration occurred in the two groups. One patient in group B had no improvement in postoperative symptoms, and the preoperative JOA score was 1. There was severe spinal cord degeneration before the operation. During the operation, the OLF was removed completely, but spinal cord function was not significantly improved. The JOA score reached 2 points at the last follow-up. One patient in group A presented with progressive aggravation of LE symptoms 12 h after surgery. Epidural haematoma was considered. After emergency debridement, hormone and dehydration drugs were administered, muscle strength gradually recovered, and the patient recovered to their preoperative level 1 month after surgery. There was cerebrospinal fluid leakage caused by dural tears in group A (*n* = 4) and group B (*n* = 12). The preoperative average JOA score of group A was 6.37 and that of group B was 5.19. In group A, the average JOA score at 6 months, 1 year and 2 years after surgery was 7.87, 8.23 and 8.26, respectively, and the average JOA score improvement rate was 32.79 %, 38.32 and 38.53 %, respectively. In group B, the average JOA score at 6 months, 1 year and 2 years after surgery was 7.74, 8.15 and 8.29, respectively, and the average JOA score improvement rate was 39.15 %, 46.86 and 47.12 %, respectively. The preoperative JOA score of group B was significantly lower than that of group A, and the preoperative symptom duration of group B was significantly higher than that of group A. However, the JOA score improvement rate in group B was higher than that in group A during postoperative follow-up, especially at 1 year of follow-up, and the difference between group A and group B was statistically significant (*P* < 0.05) (Figs. 3 and 4).

## Discussion

TOLF is a disease with a relatively low incidence, and with the advent of an ageing society, TOLF has become one of the main causes of chronic thoracic spinal cord injury [14, 15]. TOLF often has a slow onset [9], with the highest incidence among people aged 50–59, and it increases with age [16]. Nearly half of the patients complained of pain and numbness in one or both lower limbs [17], which was similar to the symptoms of lumbar disease. In our study, a total of 43 patients complained of lower limb pain, and 52 patients complained of lower limb numbness. Thoracic spinal stenosis is often associated with lumbar spinal stenosis or cervical spondylosis, resulting in complex symptoms and signs in patients, and early diagnosis is sometimes difficult. The incidence and pathogenesis of TOLF in the population are not clear, mainly in Asia, which is more commonly reported in Japan [6, 18, 19]. Wang et al. [20] analysed 142 patients with TOLF and found that TOLF was related to systemic ossification disease, spinal load change and ageing. The effects of conservative treatment of TOLF are poor, its treatment is mainly surgery, and there are many kinds of surgical methods. Posterior laminectomy has become the most commonly used classical operation, which can remove the OLF while completing the decompression of the compressed spinal cord, prevent the further deterioration of spinal cord function and restore it to varying degrees.

However, due to the low prevalence rate, few studies have been reported thus far, and the safety and efficacy of different surgical methods in the treatment of secondary thoracic myelopathy due to TOLF remain unclear, especially whether combined instrumentation should be used after laminectomy. Pedicle screw internal fixation was first used in the surgical treatment of spinal deformity [21]. According to a previous literature report, increased spinal mobility after laminectomy alone could cause slight traction or vibration of the injured spinal cord at the level of the OLF, which may compromise the recovery of the injured spinal cord. In addition, increased intervertebral range of motion after laminectomy alone could lead to a concentration of mechanical stress at the lesion site, which may result in re-extension of the OLF, especially at the level of the thoracolumbar junction [22, 23].

In this study, 65 patients in group B (45.8 %) who presented with gait disturbance, urination disorder, preoperative duration of symptoms, intramedullary signal change on MRI, dural ossification, residual rate of cross-sectional spinal canal area on CT, shape on sagittal MRI, and pre-mJOA showed significant differences compared with those in group A, suggesting severe thoracic myelosis [11]. High BMI might also lead to severe ossification due to increased mechanical stress and repetitive mild trauma of thoracolumbar OLF. In patients with severe OLF, the spinal cord was fragile, and minor traction or vibrations during intraoperative removal of the ligamentum flavum might lead to severe paralysis. In addition, the facet joints of the thoracic spine play an important role in maintaining the stretching, shearing, and torsion of the thoracic spine, and extensive intraoperative resection of the lamina and facet joints can lead to spinal instability and kyphosis, which are considered to be potential causes of postoperative neurological deterioration. Posterior laminectomy with instrumentation can maintain the stability of the spine after surgery, reduce the incidence of kyphosis and avoid repetitive spinal cord trauma [24, 25]. However, there were no cases of kyphosis during the follow-up period of this study. Last but not least, it has been reported that the application of instrumentation after laminectomy is good for the postoperative recovery of the injured spinal cord due to thoracic myelopathy and prevents re-extension of OLF [26, 27]. In our study, we found that the JOA score improvement rate of group B at the 1-year follow-up was significantly different from that of group A. This might be because the spinal activity of group B had little interference with postoperative spinal cord recovery. These results showed that LI had a significant clinical effect on myelopathy caused by severe OLF, especially for patients with TOLF who had extensive laminectomy.

LI for TOLF is a major developing trend. There were some limitations in this study. First, the maximum time limit of neurological recovery after OLF in thoracic vertebrae is not clear, and the follow-up period of 2 years in our study may be insufficient. Second, this study was a retrospective single-centre study with a small sample size, so we recommend a multicentre study with a large sample size to further confirm our conclusions. Despite these limitations, we believe that this study provides important guidance in clinical work, especially in the implementation of surgical procedures.

## Conclusions

Currently, there is no consensus on whether instrumentation is needed after laminectomy for TOLF. We found that, for patients with a long duration of gait disturbance, urination disorder, preoperative duration of symptoms, intramedullary signal change on MRI, dural ossification, residual rate of cross-sectional spinal canal area on CT less than 60 %, and shape on the sagittal MRI being beak and low, pre-mJOA had better clinical effects after LI as compared to those after LA, and the incidence of perioperative complications was lower.

## Data Availability

The datasets generated and analysed during the current study are availabled from the corresponding author on reasonable request.

## Abbreviations

TOLF: Thoracic ossification of ligamentum flavum
LA: Laminectomy alone
LI: Laminectomy with instrumentation
CT: Computed Tomography
MRI: Magnetic Resonance Imaging
mJOA: Modified Japanese Orthopaedic Association
